# PROMIS computerized adaptive testing is effective and efficient for outcome assessment in patients with wrist or hand fractures

**DOI:** 10.1177/17531934261420039

**Published:** 2026-02-12

**Authors:** Michiel AJ Luijten, Pascal FW Hannemann, Lotte Haverman, Martijn Poeze, Diederik O Verbeek

**Affiliations:** 1 Amsterdam UMC, Vrije Universiteit, Department of Epidemiology and Data Science, the Netherlands; 2 Amsterdam UMC location AMC, Department of Child and Adolescent Psychiatry and Psychosocial Care, Emma Children’s Hospital, the Netherlands; 3 Amsterdam Public Health Research Institute, Methodology and Mental Health, the Netherlands; 4 Department of Surgery, Division of Trauma, Maastricht University Medical Center +, the Netherlands; 5 Amsterdam Public Health Research Institute, Mental Health and Digital Health Amsterdam, the Netherlands; 6 Trauma Section, Department of Surgery, Dijklander Ziekenhuis, the Netherlands

**Keywords:** CAT, patient-reported outcomes, PROMIS, upper-extremity, wrist-hand fracture

## Abstract

**Introduction:**

Various patient-reported outcome measures (PROMs) are available to assess functional outcomes in patients treated for wrist-hand fractures. Among the most frequently applied instruments are the Patient-Rated Wrist/Hand Evaluation (PRWHE), Quick Disabilities of the Arm, Shoulder and Hand and Short Musculoskeletal Function Assessment. Computerized adaptive tests (CATs) utilizing the PROM-Information System (PROMIS) offers a standardized alternative. This study compared the psychometric properties of PROMIS CATs with these three legacy instruments and assessed independent factors associated with worse PROMIS scores.

**Methods:**

Patients treated for wrist–hand fractures were recruited in a Level I trauma centre. Construct validity was assessed by correlating instruments (Pearson’s *R* > 0.7 was considered sufficient). Reliability (standard error (SE), *α*), efficiency (items/time needed to completion) and floor/ceiling effects were assessed per instrument. A mean SE < 2.2 was deemed sufficient and an *α* > 0.7. Factors associated with worse PROMIS scores were also identified.

**Results:**

Correlations between PROMIS measures and legacy instruments were high (*r* = 0.74–0.84), except for a moderate correlation between PROMIS-Physical Function and PRWHE (*r* = 0.63). Reliability for all PROM’s was sufficient (SE 2.1–2.2, *α* 0.92–0.97). PROMIS required fewer items (4–8 vs. 11–46) and less time (51–84 vs. 131–314 seconds). The PROMs did not exhibit floor/ceiling effects. An elevated level of depression was one of the strongest factors independently associated with worse PROMIS scores.

**Conclusion:**

PROMIS CATs showed strong correlations with legacy instruments, supporting its ability to provide comparable functional assessments. Computerized adaptive tests maintain high reliability while offering greater efficiency than legacy instruments. Elevated depressive symptoms emerged as one of the strongest independent predictors of worse CAT scores in this study.

**Level of Evidence:**

Diagnostic study, Level II

## Introduction

Outcome assessment in orthopaedic trauma has shifted from focusing solely on clinical or radiographic variables to incorporating patients’ perspectives (Makhni et al., 2023; Porter, 2010). This change reflects a growing recognition that measures such as range of motion or fracture healing do not fully capture the impact of an injury on patients’ daily functioning, quality of life or return to meaningful activities. Therefore, patient-reported outcome measures (PROMs) play a central role in complementing traditional clinical and radiographic outcomes, offering a more comprehensive understanding of recovery in patients with orthopaedic trauma.

A wide range of PROMs are commonly used in patients with upper extremity trauma (Makhni et al., 2017). Some instruments assess overall physical function, such as the Short Musculoskeletal Function Assessment (SMFA), whereas others target upper-extremity-specific function (Quick Disabilities of the Arm, Shoulder and Hand, QuickDASH) or wrist–hand-specific disability (Patient-Rated Wrist/Hand Evaluation (PRWHE)) (Beaton et al., 2005; MacDermid, 1996; MacDermid et al., 1998; Swiontkowski et al., 1999). However, many of these legacy instruments have notable limitations, including suboptimal psychometric properties and lengthy completion times, which may reduce their utility in clinical and research settings (Bell et al., 2023; Hung et al., 2013; Ziedas et al., 2022).

The Patient-Reported Outcomes Measurement Information System (PROMIS^®^) is an initiative funded by the US National Institutes of Health that aims to provide reliable, precise and standardized tools for assessing patient-reported health status across diseases, conditions and populations (Cella et al., 2007). PROMIS is offered in Dutch through the Dutch–Flemish PROMIS National Center, which is officially designated by the PROMIS Health Organization as the central information and distribution platform for PROMIS in the Netherlands and Flanders. PROMIS instruments comprise an item bank designed to capture how people feel and function in their everyday lives, focusing on symptoms (e.g. pain, depression and anxiety) and functioning (e.g. physical, mental and social health). Item banks of particular relevance to patients with upper extremity fractures include the Physical Function (PROMIS-PF), Upper Extremity (PROMIS-UE) and Pain Interference (PROMIS-PI) (Makhni, 2019; Papuga et al., 2017; Verbeek et al., 2021).

In general orthopaedics, PROMIS measures compare favourably with legacy PROMs, especially when administered as computerized adaptive tests (CATs) (Brodke et al., 2016; Fidai et al., 2018). These tests are based on the item response theory and electronically select the most relevant items for each respondent. Prior research suggests that they measure the same or similar constructs as legacy instruments while substantially reducing completion time and respondent burden compared with fixed-length questionnaires (Brodke et al., 2016; Fidai et al., 2018). Despite these advantages, PROMIS CATs remain underused in orthopaedic trauma and further psychometric evaluation, particularly in injury-specific cohorts, is needed to guide their implementation in clinical practice (Jayakumar et al., 2018; O’Hara et al., 2020).

The primary objective of this study was to correlate PROMIS (-UE, -PF and -PI) CATs to legacy instruments (PRWHE, QuickDASH and SMFA), in patients with wrist or hand fractures. The secondary objective was to assess the reliability, efficiency and floor/ceiling effects. It was hypothesized that PROMIS CATs would show strong correlations and reliability while being more efficient (in terms of the number of items administered and completion time) and that CATs would not exhibit floor or ceiling effects. Additionally, independent (sociodemographic and clinical) factors associated with PROMIS (-UE,-PF,-PI) CAT scores were assessed.

## Methods

### Design

This prospective, cross-sectional, single-centre study was approved by the Institutional Review Board. Patients were recruited from the orthopaedic trauma outpatient clinic of a Level I academic trauma centre over a 21 month period (1 October 2021 to 1 July 2023).

### Patients

Patients were eligible for inclusion if they had received operative or non-operative treatment for a wrist or hand fracture and had a minimum follow-up of 1 month. The exclusion criteria were age under 18 years, multiple injuries, cognitive impairment or language barriers that prevented the completion of the questionnaires in Dutch.

Eligible participants were identified from the daily clinic appointment list by one of the investigators (DV) and verbal and written study information was provided to potential candidates by the treating physician. Informed consent was obtained from patients who agreed to participate, after which they received instructions on using a wireless touchscreen tablet (Apple iPad, Cupertino, CA, USA) to complete the study questionnaires.

Of the 172 eligible patients, 56 (33%) agreed to participate.

### Data collection

Patient data and PROMs, including PROMIS CATs, PRWHE, QuickDASH and SMFA, were collected through an online PROM portal (‘KLIK’) accessible via a computer tablet (van Oers et al., 2021). To compare efficiency, the number of items and time required for completion were electronically recorded for each instrument.

### Patient characteristics

Sociodemographic and fracture-related information were collected from all patients and their electronic health records. Patients were also asked to rate their current pain intensity using the following categories: no pain, slight pain, moderate pain, severe pain or extreme pain.

### Measures

#### PROMIS CATs

PROMIS was developed using the item response theory to provide unidimensional measurement scales supported by item banks. A key advantage of this approach is that new items or items from other instruments assessing the same domain can be placed on the same metric. The Dutch–Flemish PROMIS item banks for Upper Extremity (version 2.0), Physical Function (version 1.2) and Pain Interference (version 1.1) include 46, 121 and 40 items, respectively (Crins et al., 2018, 2015; Kaat et al., 2019; Lameijer et al., 2020). The Upper Extremity Bank focuses specifically on arm function (shoulder, elbow, wrist and hand) and assesses activities of daily living and self-care (e.g. opening a door, combing hair and brushing teeth). The Physical Function Bank extends this scope to include more general lower-extremity and axial (neck and back) functions (e.g. climbing stairs and walking). The Pain Interference Bank assesses the extent to which pain limits participation in activities such as work, recreation and social life. All PROMIS items were rated on a five-point Likert scale.

PROMIS item banks were administered as CATs. Each CAT was programmed to stop once a standard error (SE) of ⩽2.2 (corresponding to 95% reliability) was reached or after a maximum of 12 items, with a minimum of two items required. PROMIS CAT scores were reported as *t*-scores (mean 50, SD 10) based on US population norms, using default US item variables (Terwee and Roorda, 2022). Higher scores indicate better physical function or more pain interference.

In addition, we assessed depression by administering the PROMIS Depression CAT (version 1.0, consisting of 28 items), comprising a variable selection of items for each patient (Flens et al., 2017).

#### PRWHE

The PRWHE is a questionnaire that addresses pain and physical function in wrist and hand conditions (MacDermid, 1996; MacDermid et al., 1998). It includes 15 items, five on pain and 10 on function. The items address specific functions and aspects of daily living (e.g. using a knife and buttoning a shirt). Each item is scored from 0 to 10. The pain and function scores were combined to form a total score ranging from 0 to 100. A lower score indicates less pain and better function, whereas a higher score reflects greater pain and disability.

#### QuickDASH

The QuickDASH is an 11-item questionnaire developed to assess upper-extremity-specific disability (Beaton et al., 2005; Kennedy et al., 2013). The items cover daily activities (e.g. opening a jar, washing and using a knife), symptoms such as pain, and social and work-related functioning. Each item was rated on a five-point Likert scale. Scores were calculated by summing item responses and converting them to a 0–100 scale, where 0 indicates no disability and 100 indicates the most severe disability.

#### SMFA

The SMFA is a 46-item questionnaire that assesses overall physical functioning, with items scored on a five-point Likert scale (Reininga et al., 2012; Swiontkowski et al., 1999). It comprises two indices: the Dysfunction Index (34 items), which assesses the perceived difficulty and frequency of problems in performing daily activities, and the Bother Index (12 items), which captures the extent to which patients are bothered by functional limitations in areas such as work and recreation. Scores are calculated by summing item responses and converting them to a 0–100 scale, with higher scores reflecting poorer function.

### Analyses

#### Correlation

Pearson correlations between PROMIS (-UE, -PF and -PI) CATs, PRWHE, QuickDASH and SMFA were calculated. A high correlation (*r* > 0.70) was expected between PROMIS-UE CAT, PRWHE and QuickDASH, given that these instruments exclusively assess wrist–hand and upper-extremity functioning (Kaat et al., 2019). A high correlation (*r* > 0.70) was also expected between the PROMIS-PF CAT and SMFA, as both instruments assess more general physical functioning (Morgan et al., 2015). Furthermore, at least moderate correlations (*r* > 0.50) were expected between PROMIS-UE CAT and SMFA, and PROMIS-PF CAT and PRWHE and QuickDASH (Havermans et al., 2023; Overbeek et al., 2015).

#### Reliability

Reliability in this study was assessed using a single measurement, reflecting internal consistency, which is the degree to which items within a questionnaire or subscale are interrelated, indicating whether they measure the same underlying constructs. For PROMIS, each *t*-score is accompanied by a standard error (SE); the mean SE was calculated for the entire sample to assess the reliability of PROMIS (-UE, -PF and -PI) CATs. A low standard error indicates high precision and therefore high reliability. An SE ⩽ 2.2 corresponds to a reliability of 0.95 and is considered sufficient.

For legacy instruments (PRWHE, QuickDASH and SMFA), internal consistency was assessed using Cronbach’s *α*, with values >0.70 regarded as indicative of sufficient reliability (Terwee et al., 2007).

#### Efficiency

Efficiency was defined as the total number of items and the time (seconds) needed for test completion. Completion times were compared using the independent-samples *t*-test and *p* < 0.05 was considered statistically significant.

#### Floor and ceiling effects

Scores were assessed for floor and ceiling effects, reflecting the lowest and highest possible health states, respectively. An instrument was considered to exhibit a floor or ceiling effect if more than 15% of the respondents achieved the minimum or maximum score across all administered items. (Terwee et al., 2007).

#### Associated factors

Univariable and multivariable linear regression analyses were performed to identify factors with the strongest association with worse PROMIS (-UE, -PF and -PI) CAT scores (corresponding with lower UE, PF and higher PI scores). The variables included in the analysis were all sociodemographic and fracture specific factors as well as depressive symptoms (as measured using PROMIS Depression CATs) and pain intensity. Variables with *p* < 0.10 in the univariable analyses were subsequently included in the multivariable linear regression analysis but education level, employment and follow-up duration were not included. No multicollinearity was detected among the included variables (*r* < 0.8). We also inspected the assumptions of normality and linearity through histograms, probability–probability plots, residual plots and scatter plots, and all assumptions for performing linear regression analysis were met.

Standardized regression coefficients (*β*) were reported to indicate the relative strength of the associations between the independent variables and PROMIS scores, with larger absolute *β* values reflecting stronger associations. The adjusted *R*² was calculated to assess the overall explanatory power of the multivariable regression models for the PROMIS CAT scores.

## Results

### Study population

The mean age of the study population of 56 patients was 49 (SD 18; range 18–76) years. The majority were men (55%), had sustained a fracture of the wrist (77%) and had received operative fracture fixation (77%) (Tables 1 and 2). The mean follow-up after treatment was 161 (SD 171; range 30–830) days.

**Table 1.** Sociodemographic variables (*n* = 56) (%).

Mean age, years (SD; range)	49 (18; 18–76)
Gender	
Male	31 (55)
Female	25 (45)
Highest education level	
Lower	8 (14)
Intermediate	31 (55)
Higher	17 (31)
Employment status	
Paid employment	23 (41)
Other	33 (59)
Social status	
Married/living with partner	37 (66)
Single/separated/widow	12 (21)
Living with parents	7 (13)

**Table 2.** Fracture specifics (*n* = 56) (%).

*Fracture location*	
Wrist	43 (77)
Hand	13 (23)
*Treatment*	
Operative	43 (77)
Non-operative	13 (23)
Mean follow-up, days (SD; range)	161 (171; 30–830)

### Correlation

Correlations between PROMIS (-UE, -PF, -PI) CATs measures and legacy instruments (PRWHE, QuickDASH, SMFA) were high (*r* = 0.74–0.84), except for a moderate correlation between PROMIS-PF CAT and PRWHE (*r* = 0.63) (Table 3). This was consistent with the *a priori* defined hypothesis.

**Table 3.** Correlation (Pearson coefficients).

Instruments	PRWHE	Quick DASH	SMFA
PROMIS-Upper Extremity CAT	0.74	0.84	0.74
PROMIS-Physical Function CAT	0.63	0.75	0.76
PROMIS-Pain Interference CAT	0.75	0.74	0.76

### Reliability

Reliability for all PROMIS CATs and legacy instruments was very high, as shown by mean SE values of 2.2, 2.1 and 2.1 for PROMIS-UE, PROMIS-PF and PROMIS-PI CATs, respectively, and Cronbach’s *α* values of 0.96, 0.92 and 0.97 for PRWHE, QuickDASH and SMFA, respectively.

### Efficiency

The mean numbers of items needed for test completion for the PROMIS-UE, PROMIS-PF and PROMIS-PI CATs were 8 (SD 3; range 4-12), 6 (SD 3; range 3–12) and 4 (SD 3; range 2–12), compared with the fixed 15, 11 and 47 items for the PRWHE, QuickDASH and SMFA, respectively. The time to completion for all PROMIS-CATS was shorter than that for all legacy instruments (*p* < 0.001 for all time intervals) (Table 4).

**Table 4.** Number of items and time to complete.

Instruments	Items; mean (SD)	Time; seconds (SD)
PROMIS-Upper Extremity CAT	8 (3)	75 (28)
PROMIS-Physical Function CAT	6 (3)	84 (40)
PROMIS-Pain Interference CAT	4 (3)	51 (31)
PRWHE	15	131 (53)
Quick DASH	11	153 (63)
SMFA	46	314 (118)

### Floor and ceiling effects

None of the PROM instruments showed floor or ceiling effects that exceeded the 15% threshold (Table 5).

**Table 5.** Floor and ceiling effects (*n* = 56) (%).

Instruments	Floor	Ceiling
PROMIS-Upper Extremity CAT	0 (0)	0 (0)
PROMIS-Physical Function CAT	0 (0)	0 (0)
PROMIS-Pain Interference CAT	0 (0)	0 (0)
PRWHE	0 (0)	4 (7)
Quick DASH	0 (0)	4 (7)
SMFA	0 (0)	1 (2)

### Associated factors

In the multivariable regression analysis, the strongest independent factor correlated with worse (lower) PROMIS-UE CAT scores was higher pain intensity, followed by elevated depressive symptoms (Table 6). For both PROMIS-PF and PROMIS-PI CAT scores, elevated depressive symptoms emerged as the strongest associated factor, followed by a higher pain intensity. The full models explained 45, 4% and 61% of the variance in the PROMIS-UE, PROMIS-PF and PROMIS-PI CAT scores, respectively.

**Table 6.** Associated factors (multivariable linear regression analysis).

Associated factors	PROMIS CATs					
	Upper Extremity		Physical Function		Pain Interference	
	*β* Coefficient	*p*-Value	*β* Coefficient	*p*-Value	β Coefficient	*p*-Value
Mean age; years	−0.151	0.232	−0.183	0.144	×	×
Gender; male (y/n)	0.096	0.452	0.114	0.358	×	×
Fracture; distal radius (y/n)	−0.152	0.163	×	×	×	×
Treatment; operative (y/n)	×	×	−0.126	0.251	0.005	0.961
Follow up; days	×	×	×	×	×	×
PROMIS-Depression	−0.309	0.014	−0.389	0.03	0.507	<0.001
Pain intensity	−0.390	0.003	−0.325	0.008	0.422	<0.001

×: not included in the analysis based on univariable analyses.

Adjusted *R*^2^ for PROMIS UE CAT: 0.450.

Adjusted *R*^2^ for PROMIS PF CAT: 0.483.

Adjusted *R*^2^ for PROMIS PI CAT: 0.613.

## Discussion

In this study, PROMIS (-UE, -PF, and -PI) CATs showed strong correlations with legacy instruments (PRWHE, QuickDASH and SMFA) with high reliability. To our knowledge, no previous studies have correlated PROMIS measures with legacy instruments in a specific cohort of patients with wrist or hand fractures. Only two studies have examined the PROMIS in a broader upper-extremity fracture population (Havermans et al., 2023; Kaat et al., 2017). Additional studies have been conducted in mixed cohorts of patients with various (traumatic and nontraumatic) upper-extremity disorders (Döring et al., 2014; Garcia-Lopez et al., 2022; Morgan et al., 2015; Overbeek et al., 2015; Phillips et al., 2019). Findings in these studies are consistent with those found in ours.

In two studies of patients with arthritic conditions of the hand, the PROMIS-UE showed moderate to high correlations (*r* = 0.43-0.65) with the PRWHE (Garcia-Lopez et al., 2022; Phillips et al., 2019). Two further studies assessed the correlation between PROMIS-UE and QuickDASH in cohorts of patients with upper-extremity trauma and mixed upper-extremity conditions, both showing strong correlations (*r* = 0.82 and *r* = 0.81) (Havermans et al., 2023; Kaat et al., 2017). In the upper-extremity trauma cohort, PROMIS-UE also showed a strong correlation with the SMFA (*r* = 0.76).

No prior studies have correlated PROMIS-PF and -PI CATs with the PRWHE. However, the PROMIS-PF has been compared with the QuickDASH, showing moderate to high correlations (*r* = 0.55–0.76) (Havermans et al., 2023; Overbeek et al., 2015). In addition, a study of proximal humerus fracture patients reported a strong correlation (*r* = 0.81) between PROMIS-PF and SMFA scores (Morgan et al., 2015). For the PROMIS-PI, previous studies in patients with upper extremity conditions found moderate to strong correlations with the QuickDASH (*r* = 0.61–0.79) across different time points (Döring et al., 2014; Havermans et al., 2023; Overbeek et al., 2015).

Computerized adaptive tests are driven by an algorithm that tailors the questionnaire to each patient by selecting only the most relevant items, thereby reducing the total number of questions administered without compromising the measurement precision. Consequently, the average completion time for all PROMIS CATs in this study was significantly shorter than that for legacy instruments. On average, CATs required approximately 1 minute to complete, representing a substantial reduction in patient burden and improving feasibility for both clinical practice and research.

In contrast to PROMIS CATs, all legacy instruments exhibited some degree of ceiling effect (up to 7%), although none surpassed the 15% threshold considered problematic. This finding is noteworthy, as ceiling effects are a well-recognized limitation of PROMs assessing upper-extremity function, particularly limiting discrimination among high-functioning patients such as young adults and athletes (Bell et al., 2023; Tyser et al., 2021). Previous studies have also reported ceiling effects with the PROMIS-UE version 1.2 item bank (Kaat et al., 2017). In contrast, our study employed version 2.0, which expanded the item bank from 15 to 46 items to enhance measurement precision [10]. This improvement probably explains the absence of ceiling effects in our results, suggesting that the PROMIS-UE v2.0 more effectively captures higher levels of functioning.

The results of this study further showed that, along with pain intensity, elevated depressive symptoms were one of the strongest independent factors associated with worse PROMIS CAT scores. Notably, more depressive symptoms were an even stronger predictor than pain intensity for the PROMIS-PI CAT, underscoring the complex interplay between psychological factors, pain perception and upper extremity impairment. These findings align with growing evidence highlighting the critical role of psychological factors and mental illness in poor outcomes among patients with orthopedic trauma (Goudie et al., 2022; Jayakumar et al., 2020; Vincent et al., 2018). Importantly, they emphasize the need to account for depressive symptoms and other psychological factors when using PROMs.

This study has several limitations. First, the results apply to patients with isolated wrist or hand injuries and may not be generalizable to those with additional injuries. Second, selection bias may have occurred, as some older patients lacking digital literacy or comfort with completing questionnaires on a tablet may have declined participation. Third, the sample size was relatively limited; however, the cohort surpassed the recommended minimum of 50 patients for most psychometric evaluations (Terwee et al., 2007). Finally, the responsiveness of PROMIS CATs in patients with hand and wrist fractures was not assessed. Although the absence of floor and ceiling effects suggests adequate score distribution, the ability of PROMIS to detect clinically meaningful change over time or to discriminate between different patient states was not assessed and warrants further study.

To conclude, PROMIS (-UE, -PF, and -PI) CATs showed strong correlations with legacy instruments (PRWHE, QuickDASH and SMFA) in patients treated for wrist or hand fractures, indicating that these measures provide comparable assessment of upper-extremity-specific and more general functioning, as well as discomfort in this population. PROMIS CATs required fewer items and significantly less time to complete than legacy instruments, while maintaining high reliability, making it more efficient and effective instruments for both clinical practice and research. Elevated depressive symptoms emerged as one of the strongest independent factors associated with worse PROMIS CAT scores, further underscoring the critical role of psychological determinants in upper-extremity disability among orthopaedic trauma patients.
